# Association between cardiopulmonary resuscitation duration and one-month neurological outcomes for out-of-hospital cardiac arrest: a prospective cohort study

**DOI:** 10.1186/s12871-017-0351-1

**Published:** 2017-04-21

**Authors:** Masahiro Kashiura, Yuichi Hamabe, Akiko Akashi, Atsushi Sakurai, Yoshio Tahara, Naohiro Yonemoto, Ken Nagao, Arino Yaguchi, Naoto Morimura, on behalf of the SOS-KANTO 2012 Study Group, Sadaki Inokuchi, Yoshihiro Masui, Kunihisa Miura, Haruhiko Tsutsumi, Kiyotsugu Takuma, Ishihara Atsushi, Minoru Nakano, Hiroshi Tanaka, Keiichi Ikegami, Takao Arai, Arino Yaguchi, Nobuya Kitamura, Shigeto Oda, Kenji Kobayashi, Takayuki Suda, Kazuyuki Ono, Naoto Morimura, Ryosuke Furuya, Yuichi Koido, Fumiaki Iwase, Ken Nagao, Shigeru Kanesaka, Yasusei Okada, Kyoko Unemoto, Tomohito Sadahiro, Masayuki Iyanaga, Asaki Muraoka, Munehiro Hayashi, Shinichi Ishimatsu, Yasufumi Miyake, Hideo Yokokawa, Yasuaki Koyama, Asuka Tsuchiya, Tetsuya Kashiyama, Munetaka Hayashi, Kiyohiro Oshima, Kazuya Kiyota, Yuichi Hamabe, Hiroyuki Yokota, Shingo Hori, Shin Inaba, Tetsuya Sakamoto, Naoshige Harada, Akio Kimura, Masayuki Kanai, Yasuhiro Otomo, Manabu Sugita, Kosaku Kinoshita, Takatoshi Sakurai, Mitsuhide Kitano, Kiyoshi Matsuda, Kotaro Tanaka, Katsunori Yoshihara, Kikuo Yoh, Junichi Suzuki, Hiroshi Toyoda, Kunihiro Mashiko, Naoki Shimizu, Takashi Muguruma, Tadanaga Shimada, Yoshiro Kobe, Kazuya Nakanishi, Takashi Shiga, Takefumi Yamamoto, Kazuhiko Sekine, Shinichi Izuka

**Affiliations:** 1 Tertiary Emergency Medical Center, Tokyo Metropolitan Bokutoh Hospital, 4-23-15 Kotobashi, Sumida-ku, Tokyo, 130-8575 Japan; 2 Nihon University School of Medicine, Tokyo, Japan; 3 National Cerebral and Cardiovascular Center Hospital, Osaka, Japan; 4 Kyoto University School of Public Health, Kyoto, Japan; 5 Nihon University Surugadai Hospital, Tokyo, Japan; 6 Tokyo Women’s Medical University Hospital, Tokyo, Japan; 7 Yokohama City University Medical Center, Kanagawa, Japan

**Keywords:** Cardiopulmonary resuscitation, Emergency medical services, Out-of-hospital cardiac arrest, Patient outcome assessment

## Abstract

**Background:**

The duration of cardiopulmonary resuscitation (CPR) is an important factor associated with the outcomes for an out-of-hospital cardiac arrest. However, the appropriate CPR duration remains unclear considering pre- and in-hospital settings. The present study aimed to evaluate the relationship between the CPR duration (including both the pre- and in-hospital duration) and neurologically favorable outcomes 1-month after cardiac arrest.

**Methods:**

Data were utilized from a prospective multi-center cohort study of out-of-hospital cardiac arrest patients transported to 67 emergency hospitals between January 2012 and March 2013 in the Kanto area of Japan. A total of 3,353 patients with out-of-hospital cardiac arrest (age ≥18 years) who underwent CPR by emergency medical service personnel and achieved the return of spontaneous circulation in a pre- or in-hospital setting were analyzed. The primary outcome was a 1-month favorable neurological outcome. Logistic regression analysis was performed to estimate the influence of cardiopulmonary resuscitation duration. The CPR duration that achieved a cumulative proportion >99% of cases with a 1-month neurologically favorable outcome was determined.

**Results:**

Of the 3,353 eligible cases, pre-hospital return of spontaneous circulation was obtained in 1,692 cases (50.5%). A total of 279 (8.3%) cases had a 1-month neurologically favorable outcome. The CPR duration was significantly and inversely associated with 1-month neurologically favorable outcomes with adjustment for pre- and in-hospital confounders (adjusted odds ratio: 0.911, per minute, 95% CI: 0.892–0.929, *p* < 0.001). After 30 min of CPR, the probability of a 1-month neurologically favorable outcome decreased from 8.3 to 0.7%. At 45 min of CPR, the cumulative proportion for a 1-month neurologically favorable outcome reached >99%.

**Conclusions:**

The CPR duration was independently and inversely associated with 1-month neurologically favorable outcomes after out-of-hospital cardiac arrest. The CPR duration required to achieve return of spontaneous circulation in >99% of out-of-hospital cardiac arrest patients with a 1-month favorable neurological outcome was 45 min, considering both pre- and in-hospital settings.

## Background

Out-of-hospital cardiac arrest (OHCA) is an important public health problem worldwide. Approximately 330,000 individuals in the United States and 275,000 individuals in Europe experience OHCA each year [1]. The survival rate for OHCA remains poor, at approximately 10% [1, 2]. The cardiopulmonary resuscitation (CPR) duration is independently and inversely associated with favorable neurological outcomes and survival [3, 4]. The optimal pre-hospital CPR duration for OHCA has been reported to be 35–48 min, after which the probability of survival falls to <1% [5, 6].

The CPR duration is probably the most important factor in the termination of resuscitation for OHCA. However, the 2010 CPR guidelines for the termination of resuscitation do not include the optimal CPR duration [7–9]. In every emergency medical service (EMS) system, patients suffering from OHCA are declared dead in the field by EMS personnel after a prescribed CPR duration has been exceeded (often 30 min) [10]. Therefore, it is very important to estimate how long CPR should be continued. However, most prior studies have focused on CPR duration in a pre-hospital setting and do not include CPR duration in a hospital setting. In urban areas, most OHCA patients are transported to emergency hospitals within the timeframe of the reported optimal pre-hospital CPR duration. Therefore, an optimal CPR duration that includes in-hospital circumstances is needed [5, 6, 11, 12]. In addition, the confounding factors both outside and inside the hospital should be assessed.

The aim of the present study was to evaluate the relationship between CPR duration (including both pre- and in-hospital settings) and neurologically favorable outcomes 1-month after cardiac arrest, adjusting for out-of-hospital and in-hospital confounders, using data from the Survey of Survivors after Cardiac Arrest, collected in the Kanto Area of Japan in 2012 (SOS-KANTO 2012).

## Methods

### Study design and setting

The SOS-KANTO 2012 is a prospective, multi-center observational study of patients with OHCA who were transported to 67 emergency hospitals in the Kanto area of Japan. The study was conducted between January 2012 and March 2013. The design and data collection methods for the SOS-KANTO 2012 have been reported in detail in previous studies [13–15]. The Kanto region consists of primarily urban areas, including the capital (Tokyo).

### Japanese emergency medical service system

The Japanese EMS system has been explained in several previous studies [13, 16]. Briefly, the EMS system is operated by each municipal government and is supervised by the Fire and Disaster Management Agency of Japan. All EMS personnel are trained to perform CPR according to the Japanese resuscitation guidelines produced by the Japan Resuscitation Council based on a statement from the International Liaison Committee on Resuscitation. The ambulance crew consists of three EMS personnel, including at least one emergency lifesaving technician (ELST). ELSTs can perform several resuscitative methods under the supervision of an online medical control, including the operation of a semi-automated external defibrillator and insertion of a supraglottic airway device. Specially trained ELSTs have been able to perform endotracheal intubation since 2004, and have been able to administer adrenaline intravenously under the supervision of an online medical control since 2006. EMS personnel in Japan are not legally permitted to terminate resuscitation in the field and all OHCA patients are transported to hospitals, except in cases where death is certain [17].

### Participants

All OHCA patients transported by EMS personnel to participating institutions during the study period were eligible for inclusion in the SOS-KANTO 2012 [13]. The present study included adult OHCA patients (18 years of age or older) who underwent CPR by EMS personnel and had a return of spontaneous circulation (ROSC) in either the pre- or in-hospital setting. Of these, cases with the initial resuscitation performed inside the hospital or missing data for onset location were excluded. In addition, cases with an unknown outcome were excluded.

### Data collection and definition

The EMS personnel collected pre-hospital information based on the Utstein-style template, including age, sex, witnessed arrest, bystander CPR, time interval, initial rhythm monitored, and pre-hospital management (intravenous adrenaline administration, advanced airway management, and shock delivery) [18]. Physicians collected data regarding in-hospital treatments (drug administration, shock delivery, targeted temperature management, coronary angiography, percutaneous coronary intervention, and use of circulatory assist devices) and outcomes. CPR duration was defined as the period from the initiation of CPR by EMS personnel to a ROSC.

### Outcome measurements

The primary end point was 1-month survival with a neurologically favorable outcome after cardiac arrest, defined as a Glasgow-Pittsburgh cerebral-performance category of 1 (good performance) or 2 (moderate disability) on a five-category scale [19]. The other Glasgow-Pittsburgh categories, including 3 (severe disability), 4 (vegetative state), and 5 (death), were defined as a 1-month neurologically unfavorable outcome. The secondary endpoint was 1-month survival after cardiac arrest.

### Statistical analysis

Descriptive statistics were calculated for all variables of interest. Continuous variables are reported using median and interquartile ranges, whereas categorical variables are summarized using counts and percentages. A multivariate logistic regression analysis for 1-month neurologically favorable outcomes and survival was performed, controlling for the following pre-determined potential confounding factors: age, sex, initial cardiac rhythm (ventricular fibrillation, pulseless ventricular tachycardia, pulseless electrical activity, or asystole), witness, bystander CPR (layperson-initiated or EMS-personnel-initiated), time interval from the call to response, pre-hospital ROSC, cardiac etiology of arrest, pre-hospital managements (shock delivery [layperson or EMS personnel], adrenaline administration, and advanced airway management), and in-hospital managements (anti-arrhythmic drug administration, targeted temperature management, coronary angiography, and use of circulatory assist devices [extracorporeal membrane oxygenation or intra-aortic balloon pumping]). Crude and adjusted odds ratios (ORs) and 95% confidence intervals (CIs) were calculated using CPR duration as both an ordinal and continuous variable. As an ordinal variable, CPR duration was classified into 5 categories: 0–10, 11–20, 21–30, 31–40, and ≥41 min. All reported *p* values are two-tailed, and values less than 0.05 were considered statistically significant.

The dynamic probability of either 1-month favorable neurologic outcome or 1-month survival was described as a function of CPR duration and defined by the proportion of the remaining patients with 1-month neurologically favorable outcomes and 1-month survival relative to all eligible patients, respectively. In addition, the cumulative proportion curves for 1-month neurologically favorable outcomes and 1-month survival after cardiac arrest against the CPR duration were described. CPR guidelines recommend the false positive rate of a diagnostic test for prognostication to be close to 0% [20, 21]. Therefore, the CPR duration achieving a cumulative proportion >99% of those with a 1-month survival and 1-month neurologically favorable outcome was determined as in prior studies [5, 22]. Statistical analyses were performed using IBM SPSS for Mac Version 23.0 (IBM Corp., Armonk, NY).

## Results

### Patient enrollment

**Fig. 1:**
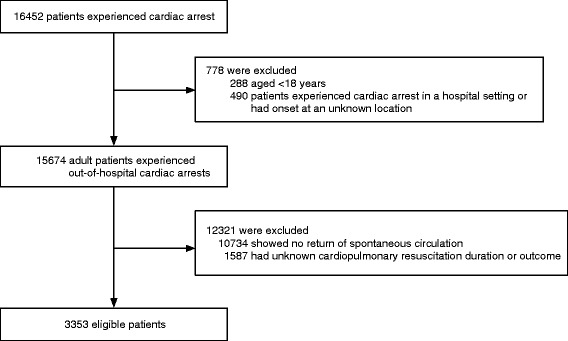
Flow diagram for patient selection

During the study period, a total of 16,452 patients with cardiac arrest were registered in the SOS-KANTO 2012. Among the 15,674 adult OHCA cases, 10,734 never obtained a ROSC in or out of the hospital. After excluding cases with unknown CPR duration or outcome, 3,353 cases were finally enrolled in the present study (Fig. 1).

### The characteristics and outcomes of the enrolled patients

The patient characteristics and outcomes are shown in Table 1. The median CPR duration was 30.0 min (interquartile range, 20.0–38.0 min). Pre-hospital ROSC was obtained in 1,692 cases (50.5%), and 1,661 cases (49.5%) obtained a ROSC in the hospital. A total of 514 (15.3%) and 279 (8.3%) cases had a 1-month survival and 1-month neurologically favorable survival, respectively.

**Table 1:** Baseline characteristics and outcomes of participants according to 1-month neurological outcomes

Characteristics	Total (*n* = 3353)	1-month neurologically favorable outcome (*n* = 279)	1-month neurologically unfavorable outcome (*n* = 3074)
Pre-hospital variables			
Age (years)	75.0 [63.0–83.0]	63.0 [53.5–71.0]	76.0 [64.0–84.0]
Male	2087 (62.2)	212 (76.0)	1875 (61.0)
Bystander CPR			
No bystander involvement	2021 (60.3)	134 (48.0)	1887 (61.4)
Layperson-initiated	870 (25.9)	80 (28.7)	790 (25.7)
EMS-personnel-initiated	462 (13.8)	65 (23.3)	397 (12.9)
Witness	2258 (67.3)	229 (82.1)	2029 (66.0)
Pre-hospital defibrillation (layperson or EMS personnel)	668 (19.9)	193 (69.2)	475 (15.5)
Advanced airway management	1651 (49.2)	73 (26.2)	1578 (51.3)
Initial shockable rhythm	492 (14.7)	184 (65.9)	308 (10.0)
Initial cardiac rhythm			
Ventricular fibrillation	483 (14.4)	182 (65.2)	301 (9.8)
Pulseless ventricular tachycardia	9 (0.3)	2 (0.7)	7 (0.2)
Pulseless electrical activity	1355 (40.4)	84 (30.1)	1271 (41.3)
Asystole	1506 (44.9)	11 (3.9)	1495 (48.6)
Pre-hospital adrenaline administration	812 (24.2)	28 (10.0)	784 (25.5)
Pre-hospital ROSC	1692 (50.5)	202 (72.4)	1490 (48.5)
Cardiac etiology of arrest	1573 (46.9)	227 (81.4)	1346 (43.8)
In-hospital variables			
In-hospital ROSC	1661 (49.5)	77 (27.6)	1584 (51.5)
Antiarrhythmic drug administration	325 (9.7)	65 (23.3)	260 (8.5)
Coronary angiography	415 (12.4)	173 (62.0)	242 (7.9)
Targeted temperature management	707 (21.1)	168 (60.2)	539 (17.5)
Circulatory assist device	249 (7.4)	64 (22.9)	185 (6.0)
Time intervals			
Call to response (min)	7.0 [5.0–9.0]	7.0 [5.0–8.0]	7.0 [5.0–9.0]
CPR to Arrival (min)	22.0 [16.0–27.0]	21.0 [15.0–27.0]	22.0 [16.0–27.0]
CPR duration (min)	30.0 [20.0–38.0]	9.0 [5.0–16.0]	31.0 [22.0–39.0]
Outcomes			
Survival to admission	2298 (68.5)	279 (100.0)	2020 (65.7)
1-month CPC grade			
CPC 1	217 (6.5)	217 (77.8)	0 (0.0)
CPC 2	62 (1.8)	62 (22.2)	0 (0.0)
CPC 3	94 (2.8)	0 (0.0)	94 (3.1)
CPC 4	141 (4.2)	0 (0.0)	141 (4.6)
CPC 5	2831 (84.4)	0 (0.0)	2831 (92.1)

Data are shown as medians [interquartile ranges] for continuous variables and numbers (percent) for categorical values

*CPC* cerebral-performance category, *CPR* cardiopulmonary resuscitation, *EMS* emergency medical service, *ROSC* return of spontaneous circulation

### The results of the multiple logistic regression analysis

The crude and adjusted ORs of a 1-month neurologically favorable outcome and survival in terms of the CPR duration (as both an ordinal and continuous variable) are shown in Table 2. The CPR duration was significantly and inversely associated with 1-month neurologically favorable outcomes and survival (adjusted OR: 0.911, per minute, 95% CI: 0.892–0.929, *p* < 0.001 for a neurologically favorable outcome; adjusted OR: 0.925, per minute, 95% CI: 0.912–0.938, *p* < 0.001 for survival). With CPR duration specified as an ordinal variable, the ORs for a 1-month neurologically favorable outcome and survival decreased stepwise.

**Table 2:** Multivariate logistic regression analysis of 1-month survival and 1-month neurologically favorable outcomes in terms of CPR duration

	1-month neurologically favorable outcome		1-month survival	
Variables	Crude odds ratio (95% CI)	Adjusted odds ratio (95% CI)	Crude odds ratio (95% CI)	Adjusted odds ratio (95% CI)
CPR duration (continuous variable), per minute	0.873 (0.861–0.886), *p* < 0.001	0.911 (0.892–0.929), *p* < 0.001	0.903 (0.894–0.911), *p* < 0.001	0.925 (0.912–0.938), *p* < 0.001
CPR duration (ordinal variable)				
0 to 10 min *n* = 372 (11.1%)	Reference	Reference	Reference	Reference
11 to 20 min *n* = 530 (15.8%)	0.241 (0.175–0.331), *p* < 0.001	0.402 (0.261–0.619), *p* < 0.001	0.259 (0.195–0.344), *p* < 0.001	0.287 (0.196–0.419), *p* < 0.001
21 to 30 min *n* = 837 (25.0%)	0.044 (0.028–0.068), *p* < 0.001	0.107 (0.059–0.195), *p* < 0.001	0.074 (0.054–101), *p* < 0.001	0.110 (0.070–0.172), *p* < 0.001
31 to 40 min *n* = 1006 (30.0%)	0.023 (0.013–0.039), *p* < 0.001	0.090 (0.045–0.181), *p* < 0.001	0.043 (0.030–0.060), *p* < 0.001	0.090 (0.055–0.146), *p* < 0.001
≥41 min *n* = 608 (18.1%)	0.016 (0.008–0.036), *p* < 0.001	0.044 (0.017–0.112), *p* < 0.001	0.030 (0.019–0.047), *p* < 0.001	0.043 (0.023–0.079), *p* < 0.001

Adjusted odds ratios were calculated controlling for the following factors: age, sex, initial cardiac rhythm, witness, bystander CPR, time interval from the call to response, pre-hospital return-of-spontaneous circulation, etiology of arrest, pre-hospital managements, and in-hospital managements

*CI* confidence interval, *CPR* cardiopulmonary resuscitation

### The dynamic probability of either a 1-month favorable neurologic outcome or 1-month survival

**Fig. 2:**
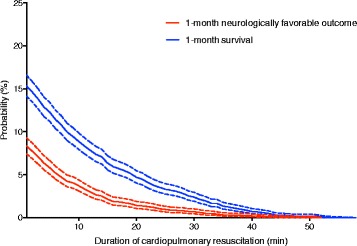
The dynamic probability of either a 1-month favorable neurologic outcome or 1-month survival relative to all patients against the duration of cardiopulmonary resuscitation. The short-dashed lines reflect the 95% confidence interval

The dynamic probability curves for a 1-month neurologically favorable outcome and 1-month survival against the CPR duration are shown in Fig. 2. After 30 min of CPR, the probability of a 1-month neurologically favorable outcome decreased from 8.3% (95% CI: 7.4–9.3%) to 0.7% (95% CI: 0.4–1.0%), and decreased from 15.3% (95% CI: 14.1–16.6%) to 2.4% (95% CI 1.9–2.9%) for survival. None of the patients who received CPR for more than 52 min survived.

### The cumulative proportion of a 1-month neurologically favorable survival in terms of CPR duration

**Fig. 3:**
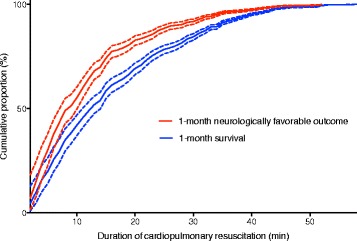
Cumulative proportion of 1-month neurologically favorable outcomes and survival against the duration of cardiopulmonary resuscitation. The short-dashed lines reflect the 95% confidence interval

The cumulative proportion curves for 1-month survival and 1-month survival with a favorable neurological outcome against the CPR duration are shown in Fig. 3. For 1-month neurologically favorable outcomes, the cumulative proportion reached >99% at 45 min of CPR (99.3%, 95% CI: 99.1–99.5%). For 1-month survival, the cumulative proportion reached >99% at 52 min of CPR (99.4%, 95% CI: 99.3–99.5%).

## Discussion

Using data from a prospective multi-center observational study, the present study demonstrated that CPR duration, including pre- and in-hospital CPR, was inversely associated with 1-month survival and neurologically favorable outcomes after OHCA, with adjustment for pre- and in-hospital confounders. In addition, the cumulative proportion of a 1-month neurological favorable outcome fell to 1% at 45 min of CPR.

CPR duration, namely ischemic time, is associated with hypo-hemodynamics and low base excess after a ROSC, which has been suggested to lead to a poor outcome [23–26]. Several studies have investigated the influence of CPR duration on survival or neurological outcomes in OHCA patients [5, 6, 11, 12, 27]. Previous studies have reported that a pre-hospital CPR duration of 26–48 min achieves a 99% cumulative proportion for neurologically favorable outcomes [5, 6, 11, 12]. In addition, the probability of survival rapidly decreases with each minute of CPR [27]. These results are consistent with those of the present study.

However, previous studies defined CPR duration in terms of CPR performed by EMS personnel in an out-of-hospital setting. Few data are available which consider both pre- and in-hospital CPR durations [27]. In population-dense areas, many patients suffering from OHCA are transported to emergency hospitals before the CPR duration has reached 30 min [28]. In the present study, the median duration of CPR to hospital arrival was 22 min, and about half of the patients achieved a ROSC in the hospital. Therefore, estimating the critical CPR duration must include consideration of pre- and in-hospital settings. Moreover, while many previous studies adjusted for pre-hospital confounders, in-hospital factors were left unadjusted [5, 6, 11, 12].

The decision to terminate resuscitation is more challenging than that for other interventions. It has been argued that a success rate of <1% still justifies the resuscitation effort [22]. Therefore, termination of resuscitation could be permissible after the CPR duration has reached the time associated with a cumulative proportion >99% for 1-month neurologically favorable outcomes or 1-month survival. Given the results of the present study, CPR could reasonably be continued for a total of 45 or 52 min, considering both the pre- and in-hospital CPR duration.

Furthermore, CPR duration is a very important factor in the consideration of advanced therapy. In advanced therapies, such as extracorporeal assisted CPR, CPR duration should be considered as a component of the protocol [4, 27, 29, 30]. For example, Reynolds et al. showed that the optimal time for considering an alternative method to conventional CPR is within 15 min [27]. In the present study, the probability of a 1-month neurologically favorable outcome rapidly decreased from 8.3 to 0.7% after 30 min of CPR. Therefore, advanced therapies should be introduced as soon as possible. Moreover, to estimate the effectiveness of these therapies, CPR duration should be taken into account.

There are several limitations in the present study. Firstly, the present study was an observational study; therefore, we could not adjust for unmeasured confounders, and resuscitation efforts might not have been conducted consistently in all OHCA patients. Secondly, we could not divide the CPR duration into out-of-hospital and in-hospital durations. In-hospital CPR duration could be a potential confounder; however, we could not include in-hospital CPR duration in the analysis because of multicollinearity. Thirdly, the SOS-KANTO 2012 was not a population-based study; it was conducted only at certain hospitals in the Kanto area of Japan. For example, the results of the present study are substantially conditioned by the call-to-response time. The observed call-to-response time of 7 min in the present study is the same as that in a nationwide study conducted in Japan [5], which may limit the generalizability of the results from these studies to other countries. Fourthly, this study excluded pediatric patients; therefore, the relevance of our findings to this population is unclear. Finally, the effect of CPR duration should be reevaluated as innovations for the pre- and in-hospital management of OHCA progress in the future.

## Conclusion

CPR duration was independently and inversely associated with 1-month survival and 1-month neurologically favorable outcomes after OHCA. The CPR duration required to achieve a ROSC in >99% of OHCA patients with a 1-month favorable neurological outcome was 45 min, considering both pre- and in-hospital settings.

## Key messages

- Longer CPR duration is independently and inversely associated with 1-month survival and neurologically favorable outcomes with adjustment for pre- and in-hospital confounders.
- The cumulative proportion of 1-month neurological favorable outcomes fell to 1% at 45 min of CPR.

## Abbreviations

CI: Confidence interval
CPC: Cerebral-performance category
CPR: Cardiopulmonary resuscitation
ELST: Emergency lifesaving technician
EMS: Emergency medical service
OHCA: Out-of-hospital cardiac arrest
OR: Odds ratio
ROSC: Return of spontaneous circulation
SOS-KANTO 2012: The survey of survivors after cardiac arrest in the kanto area in 2012
